# An *In Vivo* Quantitative Comparison of Photoprotection in *Arabidopsis* Xanthophyll Mutants

**DOI:** 10.3389/fpls.2016.00841

**Published:** 2016-06-21

**Authors:** Maxwell A. Ware, Luca Dall’Osto, Alexander V. Ruban

**Affiliations:** ^1^School of Biological and Chemical Sciences, Queen Mary University of LondonLondon, UK; ^2^Dipartimento di Biotecnologie, Università di VeronaVerona, Italy

**Keywords:** non-photochemical quenching, pNPQ, xanthophylls, photosystem II, *Arabidopsis*

## Abstract

Contribution of different LHCII antenna carotenoids to protective NPQ (pNPQ) were tested using a range of xanthophyll biosynthesis mutants of *Arabidopsis*: plants were either devoid of lutein (*lut2*), violaxanthin (*npq2*), or synthesized a single xanthophyll species, namely violaxanthin (*aba4npq1lut2*), zeaxanthin (*npq2lut2*), or lutein (*chy1chy2lut5*). A novel pulse amplitude modulated (PAM) fluorescence analysis procedure, that used a gradually increasing actinic light intensity, allowed the efficiency of pNPQ to be tested using the photochemical quenching (qP) parameter measured in the dark (qP_d_). Furthermore, the yield of photosystem II (ΦPSII) was calculated, and the light intensity which induces photoinhibition in 50% of leaves for each mutant was ascertained. Photoprotective capacities of each xanthophyll were quantified, taking into account chlorophyll *a/b* ratios and excitation pressure. Here, light tolerance, pNPQ capacity, and ΦPSII were highest in wild type plants. Of the carotenoid mutants, *lut2* (lutein-deficient) plants had the highest light tolerance, and the joint the highest ΦPSII with violaxanthin only plants. We conclude that all studied mutants possess pNPQ and a more complete composition of xanthophylls in their natural binding sites is the most important factor governing photoprotection, rather than any one specific xanthophyll suggesting a strong structural effect of the molecules upon the LHCII antenna organization and discuss the results significance for future crop development.

## Introduction

The thylakoid membrane of chloroplasts contain an array of light harvesting pigment-protein complexes, essential for oxygen evolution and photosynthesis. Plants have a highly efficient light harvesting system, with energy converting processes arranged thermodynamically to minimize energy losses (Fleming et al., 2012). Indeed, the 10^-15^ second photon capturing process by the light harvesting antenna (LHCII) of Photosystem II (PSII) is succeeded by a 10^-10^ second exciton transfer process to the PSII reaction center (RCII) (Ruban, 2012). Whilst this is highly efficient in low light, in high light conditions an energy bottleneck is formed in the thylakoid membrane, and an accumulation of excess energy arises which can result in photodamage (Ohad et al., 1984; Aro et al., 1993; Barber, 1995).

In plants, the high quantum efficiency of light harvesting at low irradiances is due to the highly conserved Lhcb gene family, which is responsible for the major (LHCII) and minor antenna protein complexes (CP24, 26, 29). LHCII exists as a trimer, with typically between two strongly, moderately and loosely bound trimers per dimeric core complex (C_2_S_2_M_2_ or C_2_S_2_M_2_L_2_) depending on the plant acclimation history (Caffarri et al., 2009). LHCII trimers are coupled to minor antenna proteins, S_2_ to CP26 and M_2_ to CP29 and CP24, which play an important role in the stability of the PSII supercomplex (Boekema et al., 1995; Caffarri et al., 2009). RCII exists as a dimer, containing D1 and D2 proteins, as well as CP43 and CP47 proteins. The absorption profiles of membrane proteins are such that an energy flow toward RCII is relatively favorable, whereupon exciton to electron energy transformation occurs, and primary electron chain acceptors are reduced. As well as binding 14 chlorophyll (Chl) molecules, each LHCII monomer binds four xanthophylls (Bassi and Dainese, 1992; Liu et al., 2004). The binding sites are labeled as L1, L2, N1, and V1, where lutein, neoxanthin, and violaxanthin are bound (Bassi and Caffarri, 2000). The location and specificity of these binding sites alludes to the importance of structural roles fulfilled by xanthophylls, but also the energy capture and transfer roles they play. Both lutein binding sites (L1 and L2) are centrally located in the LHCII monomer, with the lutein pigments both having trans-configurations, binding central helices A and B in the major LHCII antenna protein central supercoil (Plumley and Schmidt, 1987; Liu et al., 2004). Neoxanthin is located in a Chl *b*-rich region around helix C, and is the most polar of the xanthophylls (Domonkos et al., 2013). The final V1 binding site is found at the interface between connected monomeric LHCII proteins. This has been the suggested site of the interconversion between violaxanthin, antheraxanthin, and zeaxanthin. The effects of altering Arabidopsis xanthophyll stoichiometry on light harvesting protein structure and energy transfer pathways have been well documented. Croce et al. (1999a,b) reported that in recombinant LHCII, neoxanthin was essential for the binding of more than two xanthophylls to LHCII, however, occupancy of site N1 is not required for LHCII folding (Dall’Osto et al., 2006). It is now widely acknowledged that lutein is not essential for both the *in vitro* (Formaggio et al., 2001; Liu et al., 2004) as well as the *in vivo* folding of LHCII (Dall’Osto et al., 2006), while many *in vivo* reports showed that lutein is fundamental for the trimerization of LHCII (Bishop, 1996; Heinze et al., 1997; Polle et al., 2001; Lokstein et al., 2002; Havaux et al., 2004). Lutein-deficient (*lut2*) plants are, however, still able to form monomeric LHCII, and have the same chl *a*/*b* ratios as WT plants (Supplementary Table S1; Fiore et al., 2012). It is apparent that altering the xanthophyll composition of LHCII causes structural variations and changes in the excited state dynamics in the light harvesting network of PSII (see Fuciman et al., 2012 for more details).

Xanthophylls are accessory light harvesting pigments in the photosynthetic apparatus of green plants (Dall’Osto et al., 2014). Xanthophylls complement the absorption capacity of Chls in the Soret band region (Bassi and Caffarri, 2000; Fuciman et al., 2012). The collection of xanthophylls in the photosystems have a wide absorption spectrum and have higher molar extinction than Chl *a*. These traits together make them useful accessory pigments to have in the thylakoid membrane. Xanthophylls, however, have very short excited state lifetimes, approximately 500 times shorter than Chl, thus it’s thought to be effective light harvesters, they must engage in singlet-energy transfer with neighboring Chl-binding proteins (Ruban, 2012).

The final and perhaps most important role involves the photoprotective role of xanthophylls, by quenching excess energy from PSII. Non-photochemical Chl *a* fluorescence quenching (NPQ) is the readily measured assessment of the fastest underlying mechanism, energy dependent quenching (qE), employed by plants to remove this excess energy (Bilger and Björkman, 1990; van Kooten and Snel, 1990; Ruban et al., 2012; Ruban and Mullineaux, 2014). This process removes the excitation energy from PSII as heat, most likely via chlorophyll–chlorophyll and chlorophyll–carotenoid interactions. Carotenoids are potential successful energy quenchers as their S_1_ state is energetically lower than S_1_ state of Chls and is thus able to accept energy from this potentially damaging pigment (Reimers et al., 2013; Duffy and Ruban, 2015). Pioneering work by Demmig-Adams (1990) showed that qE was threefold higher in the presence of zeaxanthin, whilst simultaneously photoinhibition was reduced. It is now well documented that zeaxanthin plays many roles in the thylakoid membrane upon exposure to high light (Dall’Osto et al., 2012). Dependent on a ΔpH formation across the thylakoid membrane, zeaxanthin epoxidation occurs within seconds, yet it remains for up to an hour after light exposure (Wraight and Crofts, 1970; Krause, 1974; Demmig et al., 1987). Zeaxanthin is the most hydrophobic xanthophyll which promotes LHCII complexes clustering, whereas violaxanthin is more polar, and thus maintains the relatively high fluorescence level of LHCII (Ruban et al., 2011). The presence of zeaxanthin therefore enables the membrane to be in a standby photoprotective mode, which is one of the partially quenched states of the LHCII aggregation model (Horton and Ruban, 1992; Horton et al., 1996). If the plant is exposed to high light again, the dissipative state can be activated more quickly to minimize photodamage. Zeaxanthin achieves this by altering the relationship between ΔpH and qE. The presence of zeaxanthin shifts the lumen pH required to cause qE from 4.5–5.0 to 5.7–6.2, thus increasing the antenna affinity for protons (Rees et al., 1989, 1992; Noctor et al., 1991). Reducing the lumen acidity enables the electron transport chain to continue photochemical activity even in qE inducing conditions. Zeaxanthin, although not essential for qE, is an important factor in maximizing it (Crouchman et al., 2006; Ware et al., 2014). Lutein is also a relatively hydrophobic xanthophyll, and recently reports have emerged suggesting it has a major role in qE. This was evidenced by Niyogi et al. (1997) who demonstrated that in *Chlamydomonas reinhardtii*, α-carotene deficient mutants (*lor1*) formed less NPQ than zeaxanthin deficient mutants (*npq1*). Specific knock-out of lutein biosynthesis (*lut1*, *lut2*) in *Arabidopsis* again showed reduced NPQ compared to WT plants (Pogson et al., 1998), while higher amounts of lutein were shown to restore the qE capacity in mutants devoid of zeaxathin (Li et al., 2009). More recently the mechanism of energy dissipation by lutein have been proposed. Firstly, Ruban et al. (2007) showed that quenched LHCII transfer energy to lutein 1 via a conformational change opening a dissipative channel. In addition, Chmeliov et al. (2015) have calculated that chlorophyll-lutein energy transfer is the major dissipation pathway in a complete pigment model of LHCII, whilst simultaneously showing that violaxanthin and antheraxanthin play no role in this process.

Despite much evidence supporting the role of zeaxanthin and lutein as primary quenchers of excess light energy in plants, there has not been a direct quantitative comparison of the protective effectiveness of both xanthophylls *in vivo*. Indeed, until recently it was impossible to accurately determine the point of photoinhibition in leaves without disrupting light treatment, or without performing invasive biochemical analysis which is often difficult to quantify. Using qP_d_ as the fluorescence parameter to assess the state of RCII instead of Fv/Fm ([Fm-Fo]/Fm), offers a more accurate and versatile quantification of the state of PSII. Fv/Fm is majorly dependent on the parameter Fm, which is preferentially quenched by NPQ. As NPQ encompasses many quenching processes: energy dependent quenching (qE), zeaxanthin dependent quenching (qZ), state transitions (qT), chloroplast avoidance photorelocation (qM), and so-called photoinhibition (qI); the dark period after light exposure would have to be varied for example according to plant acclimation history, the type of mutant, the amount of zeaxanthin. However, recovery periods of 10–30 min dark are usually applied, which indeed offers some ambiguity. It is therefore difficult to distinguish between a highly protective plant with large NPQ and a photodamaged counterpart using traditional parameters, as both undermine the yield of PSII (ΦPSII). Indeed, we propose that the terms qE, qZ, qT, and qI may even be outdated and lead to uncertainty when assessing the NPQ capacity of plants. These terms have long-been characterized by their formation and relaxation speeds, yet there is considerable overlap with these, and many shared markers, such as zeaxanthin formation, trapped protons, and reorganization of LHCII trimers (Jahns and Holzwarth, 2012). qP_d_ is dependent on the difference between Fo′_act._ and Fo′_calc._ (see Materials and methods), where photoinhibition can be readily measured via changes in the measured and calculated minimum fluorescence values. Furthermore, 10 s of far red (FR) light is enough to temporarily relieve excitation pressure, thus light treatments do not need to be interrupted to gage the functionality of RCII. We have previously shown that the parameters, protective NPQ (pNPQ) and photochemical quenching in the dark (qP_d_), are the only method which can detect the earliest signs of photoinhibition, and these offer a more informative and accurate assessment of ΦPSII. They also allow for a quantification of photoinhibition and can be used to calculate the critical light intensity which causes photodamage in 50% of leaves (Ruban and Murchie, 2012; Ware et al., 2014, 2015a). Recently, Giovagnetti and Ruban (2015) have demonstrated that the qP_d_ fluorescence parameter corroborates with other methods for assessing the state of RCII, and that a qP_d_ decline directly correlates with a loss in the ΦPSII of oxygen evolution. Here we have applied these novel parameters to quantify the contribution of vital xanthophylls in PSII photoprotection and measure ΦPSII.

## Materials and Methods

### Plant Material

*Arabidopsis thaliana* Colombia-0 (Col-0) ecotype (WT), *lut2* (lacks α-carotene and lutein), *npq2* (lacks violaxanthin and neoxanthin), *lut2npq2* or *zea* (all xanthophylls are replaced by zeaxanthin), *aba4npq1lut2* or *viol* (all xanthophylls are replaced by violaxanthin) and *chy1chy2lut5* or *lute* (all xanthophylls are replaced by lutein) plants were grown on a 6:6:1 ratio of Levington M3 compost, John Innes no. 3 and perlite. Seeds were sterilized for 5 min in a mixture of 50% ethanol and 0.1% Triton-X 100 before being washed three times in distilled water. Seedlings were grown under 100 μmol photons m^-2^ s^-1^ for 1 week, and then moved to short-day conditions of 10-h at 250 μmol photons m^-2^ s^-1^, 60% humidity and 24°C. Plants were watered in trays three times per week. Biochemical and fluorescence measurements were conducted on plants from 40 to 50 days old, which showed no signs of inflorescence.

### Theory

The yield of Photosystem II (ΦPSII) is undermined by two factors: non-photochemical Chl *a* fluorescence quenching (NPQ) and the photoinhibition of RCII:

$$\varphi \mathrm{PSII}=\frac{\mathrm{qPd}.\frac{\mathrm{Fv}}{\mathrm{Fm}}}{\left[1+(1-\frac{\mathrm{Fv}}{\mathrm{Fm}}).\mathrm{NPQ}\right]}$$

Fv/Fm is the PSII maximum photochemical quantum yield; this is calculated as (Fm-Fo)/Fm, with Fm and Fo being the maximum and minimum yields of fluorescence, respectively. NPQ is calculated as (Fm/Fm’)-1. qP_d_, the coefficient of photochemistry in the dark, is calculated as:

$$qPd=\frac{\left(Fm\prime -Fo\prime act.\right)}{\left(Fm\prime -Fo\prime calc.\right)}$$

Where Fo′_act._ and Fo′_calc._ are the actual and calculated minimum levels of fluorescence in the dark after prior actinic light (AL) illumination. Fo′_calc._ is quantified according to the formula of Oxborough and Baker (1997):

$$Fo\prime calc.=\frac{1}{\frac{1}{\mathrm{Fo}}-\frac{1}{\mathrm{Fm}}+\frac{1}{Fm\prime }}$$

Under low light intensities the formula of Oxborough and Baker matches the measured Fo′ well, however, under high irradiance the two Fo′ values diverge. This is due to the rise in minimum fluorescence caused by the photo-induced permanent closure of RCII. Here, Fo′_calc._ becomes < Fo′_act._ and qP_d_ < 1.00, at this point the leaf is considered photoinhibited. When qP_d_ is > 0.98, NPQ is considered protective and called pNPQ. See Ruban and Murchie (2012) for a detailed description of the principles of the method.

### Fluorescence Measurements

Fluorescence was measured using a Walz JUNIOR-PAM (Effeltrich, Germany), 50 cm fiber-optic, magnetic leaf clip and fluorescence standard foil. Whole intact leaves from plants 40–50 days old were dark adapted for 45 min before being exposed to an increasing AL procedure. The procedure was run as a pre-programmed batch file where the scheme was: (SP)-(AL on)-(120s)-(SP)-(180s)-(SP)-(AL off/FR on)-(7 s)-(SP)-(5 s)-(AL on/FR off)-repeat. Here AL represents AL, SP the saturating pulse and FR is far red light. AL values used are 0, 90, 190, 285, 420, 625, 820, 1150, 1500 μmol photons m^-2^ s^-1^. AL intensities of 80 and 90% of these values were achieved by manually decreasing the AL intensity in the program system setting. Ten repeats for each increasing AL scheme were conducted making a total of 30 repeats for each mutant line. Only 5 repeats of each AL regime were conducted for the WT, thus totalling 15 measurements, as pNPQ assessment on WT plants grown at 250 μmol photons m^-2^ s^-1^ had been previously reported, and results were well matched (Ware et al., 2014, 2015a). AL intensities of 0, 81, 186, 281, 396, 611, 801, 1076, 1250 μmol m^-2^ s^-1^ were used on the IMAGING-PAM with the same pNPQ assessment procedure.

### Chloroplast Extraction

Whole attached leaves were dark adapted for 45 min prior to extraction. Chloroplast extraction was performed in homogenisation buffer (0.45 M Sorbitol, 20 mM Tricine pH 8.4, 10 mM EDTA, 10 mM NaHCO_3_, 0.1% BSA;) using a Polytron blender. Samples were filtered through four layers of muslin, then four layers of muslin with a cotton wool layer. Filtrates were centrifuged at 3500 × *g* for 2 min. Chloroplast pellets were resuspended in 1 ml of resuspension buffer (0.3 M Sorbitol, 20 mM Tricine pH 7.6, 5 mM MgCl_2_, 2.5 mM EDTA). Sample concentrations were calculated according to Porra et al. (1989) in 80% acetone after centrifugation for 5 min at 14,000 rpm. Final concentrations used were 35 μg/ml Chl in 1.6 ml final volumes, with samples diluted using resuspension buffer. Dark adapted samples were immediately frozen in liquid nitrogen. Illuminated samples were exposed to 850 μmol photons m^-2^ s^-1^ for 10 min using a DUAL-PAM-100 (Walz, Effeltrich, Germany), before being immediately frozen in liquid nitrogen and stored at -20°C until pigment analysis using high-performance liquid chromatography (HPLC) was performed.

### Non-denaturing Electrophoresis

Non-denaturing Deriphat-PAGE was performed following the method developed by Peter et al. (1991) with modifications described by Havaux et al. (2004). Thylakoids concentrated at 1 mg/mL chlorophylls were solubilized in a final concentration of either 0.4% dodecyl-α-maltoside (α-DM) or 1% β-DM, and 25 μg of chlorophyll were loaded in each lane. Densitometric analysis of bands’ profiles was carried out using GelPro software (Bio-Rad).

### Pigment Analysis

Chl *a/b* ratios and total Chl content were estimated by absorption spectra (Ultrospec 2100 pro spectrophotometer, GE Healthcare) in a final 80% acetone concentration. Leaf material extraction was performed according to Porra et al. (1989). Xanthophyll concentrations were determined using reverse-phase HPLC, in 100% methanol, with a LiChrospher 100 RP-18 column and Dionex Summit chromatography system (Ruban et al., 1994). Xanthophyll proportions were calculated as [mmol of a xanthophyll specie/(mmol of total xanthophylls))]. Intact chloroplasts were used for xanthophyll determination. Chloroplasts extraction and HPLC analyses were performed on three plants from each genotype, with chloroplast intactness measured by Fv/Fm.

## Results

### Quantifying the Photoprotective Capacity of NPQ and ΦPSII by PAM Fluorescence

**FIGURE 1:**
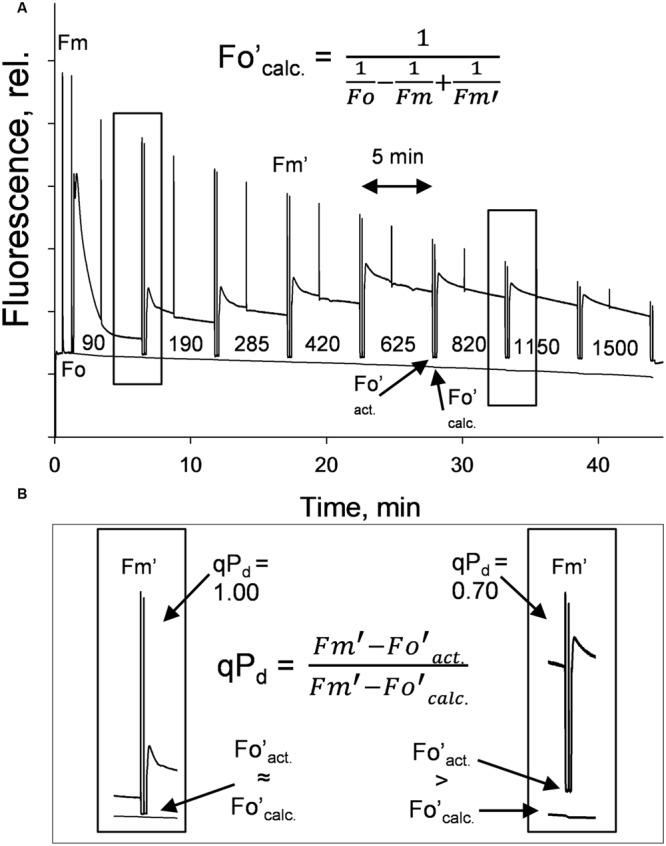
**(A)** A typical fluorescence trace from the pNPQ assessment procedure, with actinic light (AL) intensities indicated. There are eight illumination periods with each lasting approximately 5 min, with a saturating pulse in the light being applied to measure NPQ. The dark period is 10 s with far red light illumination, then a saturating pulse to measure qP_d_. **(B)** Highlights the differences between Fo′_act._ and Fo′_calc._ in high light conditions. Hundred percent of reaction centers in the left panel are open, 30% in the right are closed. Plants were dark adapted for 45 min, with 30 repeats performed on each genotype.

A gradually increasing AL procedure, representing a sunrise, was applied to whole intact leaves (**Figure 1A**), using a JUNIOR-PAM fluorometer (Walz, Effeltrich). AL values of 0, 90, 190, 285, 420, 625, 820, 1150, 1500 μmol photons m^-2^ s^-1^ were used, along with both 80 and 90% of these AL values (see Materials and Methods). NPQ was measured at 5 min intervals during AL illumination. qP_d_ results were obtained after 10 s in the dark under far red light after each incremental increase in AL (**Figure 1B**). NPQ values are considered to be pNPQ, when qP_d_ is between 0.98 and 1.00, thus ∼100% of RCII are still open in the dark after prior light exposure.

**FIGURE 2:**
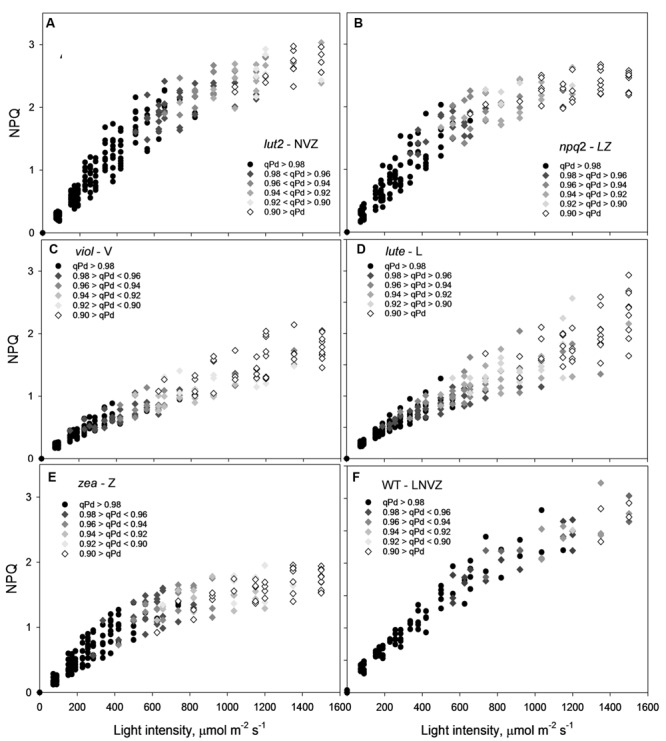
**A 3-dimensional representation of the openness of reaction centers, with the corresponding protectiveness of NPQ and AL intensity.** Black circles represent leaves where 98–100% of reaction centers are open at a particular light intensity; here NPQ is protective (pNPQ). Greyscale rhomboids represent the degree of reaction center closure (as shown on each panel). Thirty repeats were conducted for each genotype. *lut2* – no lutein, *npq2* – no violaxanthin and neoxanthin, *viol* – violaxanthin only, *lute* – lutein only, *zea* – zeaxanthin only, WT – wild type.

From the pNPQ procedure it is apparent there are different patterns of phototolerance between the mutants. Results showed statistically significant differences across the six genotypes for all parameters measured: NPQ, qP_d_ and pNPQ (ANOVA; *P* < 0.0001). Individually, *lut2* show their first signs of photoinhibition at 500 μmol photons m^-2^ s^-1^ AL (**Figure 2A**). This means that all leaves had qP_d_ values > 0.98 up until 500 μmol photons m^-2^ s^-1^ AL. This is the highest AL intensity tolerated by all leaves in any of the mutants. Furthermore, the maximum tolerated light intensity by a *lut2* leaf (where qPd >0.98) was 820 μmol m^-2^ s^-1^, which is also the highest of the mutants (**Figure 2A**). The greater proportion in the *lut2* plants is due not to the maximum NPQ level of 3.0, as the *lute* mutant can also form 3.0 NPQ, but the highest pNPQ capacity which is 2.2. This is higher than all other mutants, but 1.0 lower than the WT (**Figures 2B–F**). The other single knockout mutant, *npq2*, displays the first signs of photoinhibition at 378 μmol m^-2^ s^-1^ and maximum tolerance of 656 μmol m^-2^ s^-1^ (**Figure 2B**). *npq2* individuals have a maximum NPQ of 2.7 but importantly a maximum pNPQ of 2. The *viol* plant had the lowest pNPQ capacity of 1.1 (**Figure 2C**), despite having almost twice as much NPQ in some leaves. This manifested in the lowest light intensity which induced photoinhibition in a leaf at 152 μmol photons m^-2^ s^-1^. The *viol* mutant is also the only genotype to have all leaves with qP_d_ < 0.9 by the procedure end. This therefore illustrates that pNPQ amplitude is limited in the *viol* plants and is unable to protect at the highest light intensities, rather than just being slow to form as was seen with *npq4* genotype (Ware et al., 2014; unpublished data). The *lute* genotype is similar to the *viol* counterpart, having a low pNPQ capacity, but it has higher amounts of NPQ. This results in the first sign of photoinhibition occurring at 190 μmol photons m^-2^ s^-1^, however, it has a maximum light tolerance of 562.5 μmol photons m^-2^ s^-1^. The disparity between NPQ and pNPQ capacity is most pronounced in the *lute* mutant. Unusually the plant can form NPQ of 3, but only pNPQ of up to 1.28 (**Figure 2D**). It would therefore be interesting in future work to assess why this discrepancy occurs and how protective the remaining NPQ is, as there is less photoinhibition in this genotype, but greater NPQ than in the *viol* plant. It may be due to slower forming NPQ in the *lute* plant rather than a limit in absolute NPQ capacity, as was the case with *npq4* plants which lack PsbS in constant high light, or it could be due to changes in LHCII form and function (Ware et al., 2014). Conversely to the *lute* plant, the *zea* plant has a low NPQ capacity of 2.0 but high pNPQ maxima of 1.4. Therefore, there is much less discrepancy between the two parameters. This better pNPQ efficiency is manifested in a higher minimum photoinhibitory light intensity (285 μmol photons m^-2^ s^-1^) and maximum tolerance (738 μmol photons m^-2^ s^-1^) compared to the *lute* plant. The WT plants display the first sign of photoinhibition at 562.5 μmol m^-2^ s^-1^ and can toler 1150 μmol photons m^-2^ s^-1^. They exhibited only a marginally greater NPQ maxima than the mutants (3.2), however, a much higher pNPQ capacity of 2.8. Therefore the WT plants have the least discrepancy between NPQ and pNPQ, and better protected than the mutant plants.

**FIGURE 3:**
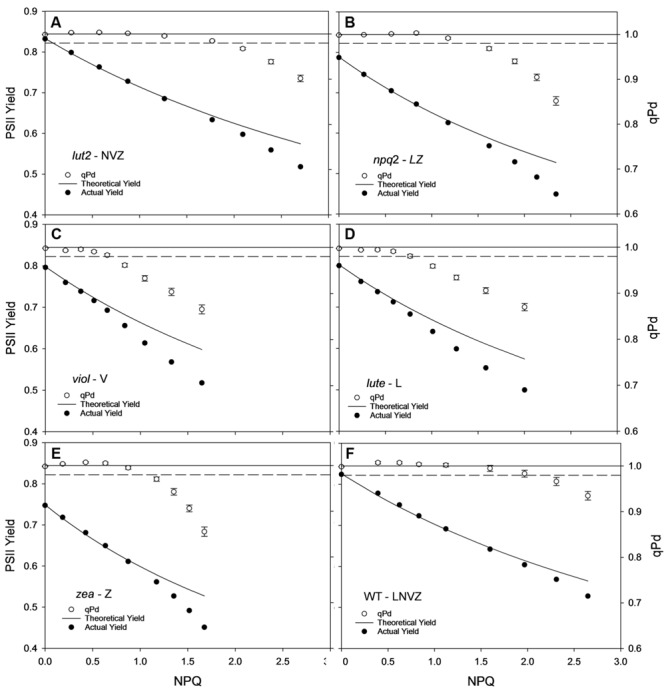
**Displays the actual (closed circles) and theoretical (black line) yields of PSII (ΦPSII) in function of NPQ and qP_d_ (open circles, see Materials and Methods) in *lut2***(A)**, *npq2***(B)**, *viol***(C)**, *lute***(D)**, *zea***(E)***Arabidopsis* mutants and the wild type **(F)** plants.** Data points are the average of 30 repeats conducted on whole intact leaves using the pNPQ assessment procedure (**Figure 1A**). Error bars represent the standard error of the mean. *lut2* – no lutein, *npq2* – no violaxanthin and neoxanthin, *viol* – violaxanthin only, *lute* – lutein only, *zea* – zeaxanthin only, WT – wild type. Theoretical PSII yield was calculated as described in Materials and Methods.

The yield of PSII (ΦPSII) is dependent on the state of reaction centers (qP_d_), pNPQ and Fv/Fm (see Materials and methods). Fundamental to the high yield of photosynthesis is the reversibility of ΦPSII downregulation. Crucially, pNPQ relaxes within seconds to minutes, yet reactivation of RCII can take many hours. Therefore, two ΦPSII are calculated here, the theoretical ΦPSII, where qP_d_ is 1.00 and pNPQ is the only antagonizing process, and the actual ΦPSII, where the state of RCII is incorporated (**Figures 3A–F**). *lut2* and *npq2* could have ΦPSII’s of 0.57 and 0.53, yet have actual ΦPSII of 0.52 and 0.45, respectively, (**Figures 3A,B**). The highest theoretical ΦPSII was 0.6 in the *viol* plants (**Figure 3C**), yet the actual ΦPSII was only 0.52. This illustrates the usefulness of ΦPSII as a parameter of fitness as both NPQ and qP_d_ have antagonistic roles on ΦPSII. The higher NPQ in *lute* plants resulted in a lower theoretical ΦPSII than *viol* plants at 0.58, and despite more open RCII, the actual ΦPSII was also lower at 0.5 (**Figure 3D**). Interestingly, the *npq2* (**Figure 3B**) and *zea* (**Figure 3E**) mutants had the lowest actual ΦPSII of 0.45, with *zea* plants only having a theoretical PSII of 0.53. Zeaxanthin expressing plants required higher light intensities to induce photoinhibition and tolerate higher maximum light intensities than *viol* and *lute* plants, yet with ΦPSII of only 0.45, they have significantly lower actual ΦPSII (t test; *P* < 0.001). As qP_d_ relaxation in the dark does not vary depending on the NPQ capacity of different mutants (unpublished data), it will take longer for zeaxanthin expressing plants to return to the maximum ΦPSII. This relationship is examined further in effects of ETR and excitation pressure (1-qP) section of the results. As well as having individuals with the highest pNPQ capacity, WT plants also had the highest actual ΦPSII with 0.53 (**Figure 3F**). High variability of Fv/Fm could affect the ΦPSII, however, in this experiment all mutants had Fv/Fm values of between 0.79 and 0.83, so the effect on ΦPSII is minimal apart from the 0.75 yield in the plants containing zeaxanthin (**Table 1**). This lower yield in the *zea* plants could possibly be explained by the presence of sustained Zea-dependent NPQ, qZ that could have some protective role.

**Table 1:** The genotype and corresponding abbreviations used in this article.

Genotype	Abbreviation	Xanthophyll composition	Fv/Fm	Chl a/b	PSI/PSII	LHCII/PSII
*lut2*	*lut2*	NVZ	0.83	3.2 ± 0.1	112 ± 19	97 ± 13
*npq2*	*npq2*	LZ	0.79	3.3 ± 0.1	73 ± 9	89 ± 14
abanpq1lut2	*viol*	V	0.80	3.8 ± 0.2	101 ± 14	97 ± 7
chy1chy2lut5	*lute*	L	0.80	4.4 ± 0.4	53 ± 11	69 ± 18
lut2npq2	*zea*	Z	0.75	3.0 ± 0.1	41 ± 11	48 ± 5
wt	*wt*	LNVZ	0.83	3.3 ± 0.0	100 ± 12	100 ± 8

*The xanthophyll composition of each plant: L, lutein; N, neoxanthin; V, violaxanthin, and Z, zeaxanthin. Fv/Fm measurements were performed on intact leaves after 45 min dark adaptation (SEM, *n* = 30 leaves). The chlorophyll *a*/*b* ratios extracted in 80% acetone according to Porra et al. (1989). (SEM, *n* = 3). PSI-LHCI, PSII core and LHCII content were evaluated upon solubilization of thylakoids with 0.4% dodecyl- α-maltoside (α-DM) and fractionation of pigment-protein complexes by non-denaturing Deriphat-PAGE. PSI/PSII and Lhcb/PSII ratios were normalized to the corresponding WT values.*

### Utilizing qP_d_ to Calculate the Leaf Population Phototolerance

**FIGURE 4:**
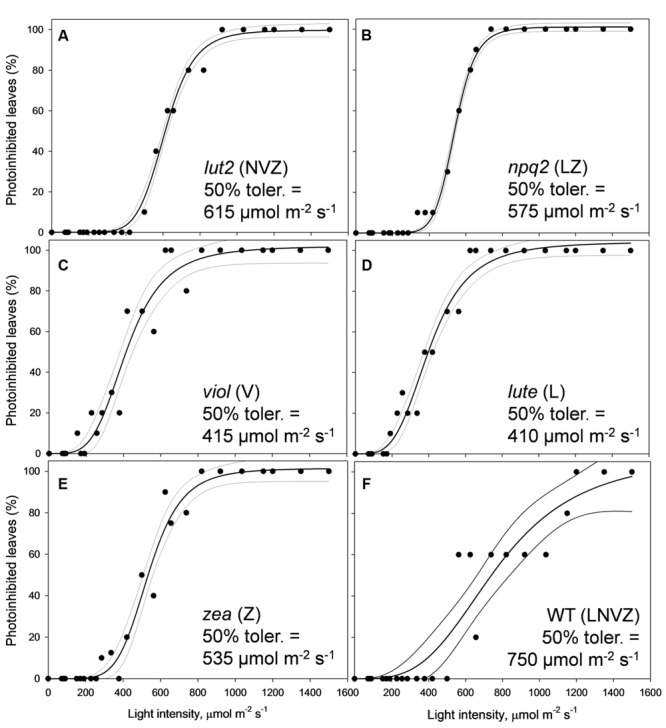
**Leaf population tolerance curves calculated using the pNPQ assessment procedure in *lut2***(A)**, *npq2***(B)**, *viol***(C)**, *lute***(D)**, *zea***(E)***Arabidopsis* mutants and the wild type **(F)** plants.** Each closed circle represents the percentage of closed reaction centers at each AL intensity. The % of photoinhibited leaves was calculated from **Figure 3** as 100 × *N*_rhombs_/*N*_total_. Regression analysis and 95% confidence intervals were performed using SigmaPlot12.0 (Sigmoidal fit, Hill 3 parameter, f = a^∗^xˆb/[cˆb+xˆb]). *lut2* – no lutein, *npq2* – no violaxanthin and neoxanthin, *viol* – violaxanthin only, *lute* – lutein only, *zea* – zeaxanthin only, WT – wild type.

**FIGURE 5:**
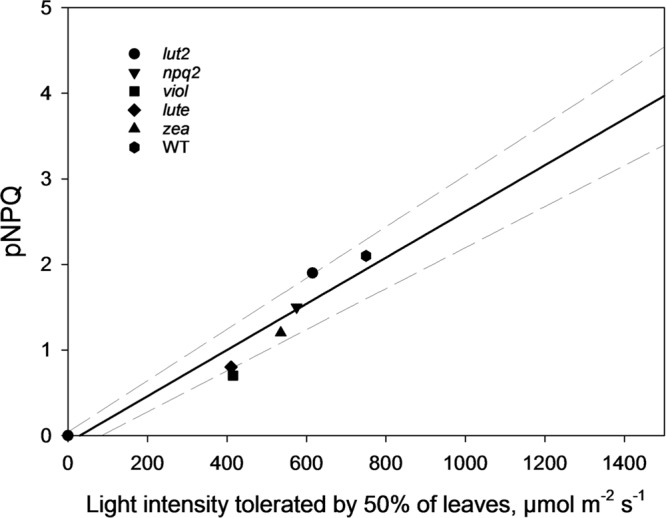
**The pNPQ value which protects each genotype, taken from **Figure 3**, and the light intensity which causes photoinhibition in 50% of leaves for that genotype.** Regression analysis was performed using SigmaPlot 12.0 (Linear, f = y0+a^∗^x). *lut2* – no lutein, *npq2* – no violaxanthin and neoxanthin, *viol* – violaxanthin only, *lute* – lutein only, *zea* – zeaxanthin only, WT – wild type.

By ascertaining the percentage of leaves which are inhibited (qP_d_ < 0.98) at each light intensity from **Figure 2** (100 × N_rhombs_/N_total_), it is possible to calculate the light intensities which cause photoinhibition in 50% of leaves. A regression analysis was then performed using SigmaPlot12.0 (Sigmoidal fit, Hill 3 parameter, f = a^∗^xˆb/[cˆb+xˆb]), from which corresponding light intensities and population tolerances can be readily extrapolated (**Figure 4**). This technique showed that the light intensities which closed RCIIs in 50% of leaves was significantly different between all genotypes (z test; *P* < 0.001%) except for between *lut2* and *npq2*, which is significant to *P* < 0.01% (*z* test), and *viol* and *lute*, which aren’t significantly different (*z* test; *P* = 0.4). WT leaves were the most tolerant, with 50% of leaves able to tolerate 750 μmol photons m^-2^ s^-1^. The next two most tolerant genotypes were from single KOs. *lut2* plants had a 50% photoinhibition point of 615 photons μmol m^-2^ s^-1^ and *npq2* 575 μmol photons m^-2^ s^-1^. Of the single xanthophyll mutants, *zea* was the most tolerant with 50% photoinhibition occurring at 535 μmol photons m^-2^ s^-1^ then *viol* and *lute* were able to tolerate 415 and 410 μmol photons m^-2^ s^-1^. One possible explanation of this relative increased tolerance in *zea* mutants could be the pre-existence of the sustained protective quenching, qZ. Therefore, it follows that phototolerance is dependent on possessing a variety of carotenoids, specifically in their particular binding sites, rather than anyone major quenching xanthophyll. Of the mutants which have one single carotenoid, the zeaxanthin only plants were better protected than lutein or violaxanthin only plants. A variety of carotenoids confers a greater pNPQ maxima, which can be clearly seen in **Figure 5**. Plotting the maximum pNPQ value against the light intensity which causes 50% of leaves to become photoinhibited illustrated that pNPQ is the determining factor for phototolerance. Furthermore, extrapolating the relationship between these points showed that a pNPQ maxima of 4.0 should be enough to protect 50% of leaves in 1500 μmol photons m^-2^ s^-1^ of light, an intensity similar to the maximum sunlight in the UK (**Figure 5**).

### Electron Transport Rates Affect the Excitation Pressure

**FIGURE 6:**
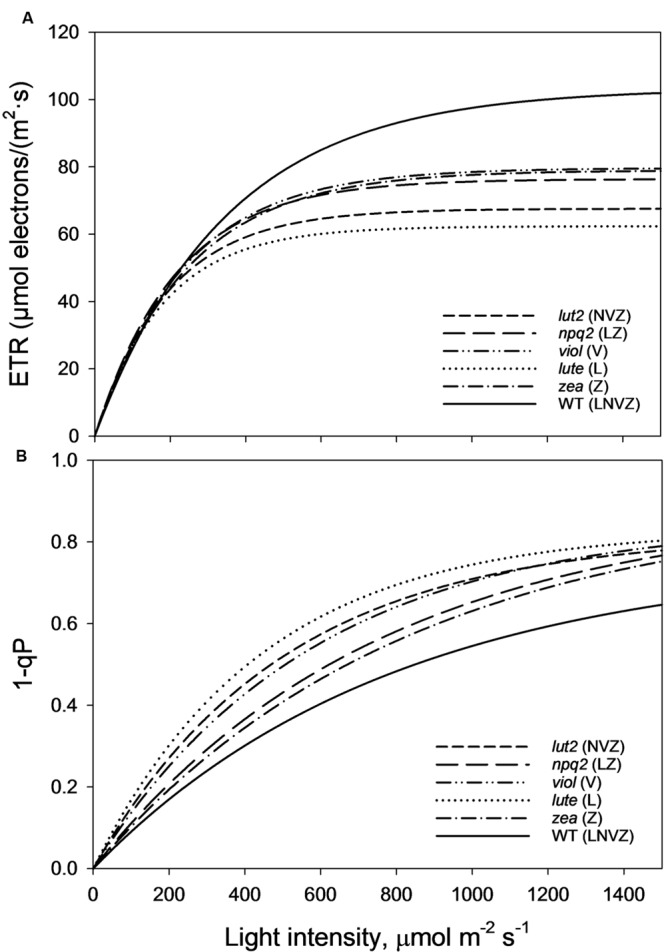
**(A) Electron transport rates (ETRs) taken from the second saturating pulse after each 5 min AL illumination period (See **Figure 1A**).** Regression analysis was performed using SigmaPlot12 (Exponential Rise to Maximum, Single 2 Parameter, f = a^∗^[1-exp(-b^∗^x)]). **(B)** Represents the excitation pressure recorded at each second saturating pulse after each 5 min AL illumination period (See **Figure 1A**). Regression analysis was performed using SigmaPlot12 (Exponential Rise to Maximum, Single 2 Parameter, f = a^∗^[1-exp(-b^∗^x)]). Thirty measurements were performed for each genotype. *lut2* – no lutein, *npq2* – no violaxanthin and neoxanthin, *viol* – violaxanthin only, *lute* – lutein only, *zea* – zeaxanthin only, WT – wild type.

As perhaps expected, owing to the well documented loss of LHCII trimeric organization in *lut2* plants (Bishop, 1996; Heinze et al., 1997; Polle et al., 2001; Lokstein et al., 2002; Havaux et al., 2004), there are different electron transport rates (ETRs) in the xanthophyll mutant plants explored here. *lut2* plants indeed had significantly reduced ETR compared to WT, *viol* and *zea* plants (*z*-test, *P* < 0.01; **Figure 6A**). The only mutant with slower ETR than *lut2* was, conversely, the lutein only plant, *lute*. Significantly slower than *lut2*, the ETR was significantly worse than all other plants (*z*-test, *P* < 0.01). Of the other three mutants, there was no significant difference between *zea*, *npq2,* and *viol* (*z*-test, *P* > 0.05). The ETR in WT plants was significantly higher than all genotypes (*z*-test, *P* < 0.05), illustrating that not only is pNPQ amplitude and light tolerance higher in the WT than all other plants, but that rates of photochemistry are too. This is due to the conserved structure of light harvesting proteins which rely on xanthophylls, not just for light absorption and dissipation, but for structural integrity.

1-qP is an effective measure of excitation pressure, and is a reflection of the combined rates of photochemistry and pNPQ. High rates of both photochemistry and pNPQ reduce the excitation pressure in the membrane. WT plants, with the highest pNPQ and greatest proportion of open RCII, had the lowest levels of excitation pressure (**Figure 6B**). The greatest difference between the WT and mutants occurs at the highest light intensity (1500 μmol m^-2^ s^-1^), where 1-qP was significantly lower than all mutants (*t*-test, *P* < 0.001). Over the course of the whole procedure, 1-qP was significantly lower in the WT than *viol*, *lute,* and *lut2* mutants (*z*-test; *P* < 0.01), but not *zea* and *npq2* plants (*z*-test; *P* > 0.05). The *zea* and *npq2* plants also have significantly less excitation pressure than *viol*, *lute,* and *lut2* plants (*z*-test; *P* < 0.01) over the course of the procedure. The mutant with the greatest excitation pressure was the *lute* plant, which has significantly higher levels than all the mutants (*z*-test; *P* < 0.05). The accumulation of excitation pressure is different between the mutants. The excitation pressure in the *lut2* and *lute* plants starts to plateau at around 1500 μmol m^-2^ s^-1^, whereas in *zea*, *viol* and *npq2* plants, there is a steeper gradient representing a greater rate of RCII closure in the light. Extrapolating this rate of closure to higher light intensities revealed that there should be less excitation pressure in the *lut2* and *lute* plants than in the *viol*/*zea* mutants (Supplementary Figure S3). Therefore, when ETR play a less prominent role, because even the WT has reached maximum ETR levels at this light intensity, the *lut2* and *lute* plants are better excess energy dissipaters than *npq2*, *viol,* and *zea* plants.

## Discussion

The results presented here offer a unique insight into the photoprotective role of xanthophylls *in vivo*. Zeaxanthin has been considered essential for NPQ since the discovery of the epoxide conversion cycle in violaxanthin, antheraxanthin and zeaxanthin xanthophylls (Sapozhnikov et al., 1957; Demmig et al., 1987; Bugos and Yamamoto, 1996). Since then it has been shown that although not essential, zeaxanthin is imperative for maximum NPQ formation (Rees et al., 1989; Niyogi et al., 1997, 1998; Ware et al., 2014). Xanthophylls have been the continued focus of the search for the ‘true quencher’ of excess energy in the photosynthetic membrane. The role of lutein has been the focus of recent research, most recently with Duffy and co-workers (Fox et al., 2015) have shown through modeling that the degrees of distortions of lutein are more important than the pigments location for quenching in the membrane. Here we addressed and quantified the photoprotective capacities of xanthophylls *in vivo* for the first time.

Using a gradually increasing AL procedure to assess pNPQ and qP_d_, it was revealed that WT plants have the highest pNPQ capacity, ΦPSII and can tolerate the highest light intensity whilst protecting 50% of leaves from photoinhibition. Of the xanthophyll mutants tested, *lut2* plants have the highest tolerance capacity, and the joint highest ΦPSII with *viol* plants. Zeaxanthin expressing plants had the highest light tolerance, yet their ΦPSII was the lowest, therefore the high NPQ levels attained were not protective at high light intensities to maintain a high ΦPSII.

WT plants and single knock-outs show that an array of xanthophylls better serves plants in photoprotection, being consistent with previous studies on sensitivity to photoxidative stress *in vivo* (Dall’Osto et al., 2012, 2013). This could be due to in part to major structural changes in light harvesting antenna, reflected in some variations in the chl *a*/*b* ratios of plants used here (**Table 1**). The non-denaturing mild electrophoresis showed certain marked differences (Supplementary Figure S1). Indeed, the LHCII/PSII ratio and PSI/PSII ratio have been affected by mutations but mainly in *zea* and *lute* plants (**Table 1**). In *zea* the LHCII antenna size decreased much stronger than in *lute* and in the both of them the relative amount of PSI decreased relatively to the control. Despite of the decreased antenna size in the both of the mutants the excitation pressure (1-qP) was higher than in the wild type. This indicates that the physical antenna size in not the only factor that governs photoinhibitory damage and hence cannot contribute greatly to the differences in the photoprotective capacity of the studied plants. Similar discrepancy between the physical antenna size and excitation pressure has been previously observed by Härtel et al. (1996) who studied the chlorine barley mutant and demonstrated that structural alterations within LHCII proteins mediated by the xanthophyll cycle are key for build-up of a large proportion of NPQ. Moreover, since the supercomplex structures were still present in our mutants it is likely that the trimer structures were present and the appearance of monomers was due to their lesser stability and resistance to the detergent. Furthermore, the high Fv/Fm values obtained here show that the plants were relatively healthy and well-acclimated so variability in fitness is likely to be negligible (**Table 1**), and HPLC analysis was well matched with previous results for xanthophyll compositions (Supplementary Table S1; Fiore et al., 2006). The enhanced photoprotection in plants with increased numbers of xanthophylls can also be due to the variability in their binding to the different LHCII domains. This variability could affect their direct quenching as well as indirect impact on the entire LHCII structure affecting its flexibility that leads to the establishment of the quenched state. It could be argued that in order to better test this, rather than using the same AL intensities for all plants to compare qP_d_ and pNPQ values, the light intensity which induces the same excitation pressure (1-qP) should be used. This would minimize the effects of reduced ETR. The problem with this method here would be that pNPQ is no longer an independent variable which can be attributed to photoprotection. Also, the relationship between pNPQ and light intensity would be redundant, and leaf population tolerance curves could not be calculated. The dissipation of excess energy as heat relieves the effective light intensity on RCII. Therefore, increasing or decreasing the AL intensity negates the beneficial effect of pNPQ. Measuring effective light intensities as 1-qP would for example be useful when measuring RCII repair, or rates of electron transport.

It is worth noting that here, it can be empirically shown that violaxanthin only plants form pNPQ. It was previously claimed that plants lacking zeaxanthin and lutein were incapable of forming NPQ (Demmig-Adams, 1990; Niyogi et al., 1997, 1998). One of the main reasons for this conclusion of a lack of NPQ could be the experimental timeframe previously employed. Typical NPQ assessment used to be performed over 5 min illumination cycles, followed by extended dark periods to assess qI and Fv/Fm. This may have not have been ample time for a slower mechanism to be instigated. Although here we too use 5 min increments for each AL intensity, the eight steps used totalled 40 min illumination, and were built on initial low light values (72–90 μmol m^-2^ s^-1^). Therefore, the total capacity of NPQ was not dependent on the speed of formation. This therefore explicitly confirmed *in vivo*, the *in vitro* demonstration that NPQ can be formed in plants lacking lutein and zeaxanthin at high ΔpH (Johnson et al., 2012).

These results lead to a number of interesting possibilities concerning the role of xanthophylls *in vivo*. Zeaxanthin expressing plants were able to delay the onset of photoinhibition the longest (**Figure 5**), yet had the lowest proportion of open RCII at the procedure end (**Figures 3** and **4**). Therefore this adds support to the consensus that zeaxanthin is important in catalyzing the aggregation of harvesting complexes, a key feature of NPQ (Ruban et al., 1991, 1994; Johnson et al., 2011; Ware et al., 2015b), particularly in the early stages of ΔpH formation. However, zeaxanthin may not have the quenching effect as big as lutein at the highest light intensities (Supplementary Figure S3). Extrapolating 1-qP curves from **Figure 6B**, *zea* and *npq2* plants would have greater excitation pressure than *viol*, *lute,* and *lut2* plants. Coupled with this, lutein and violaxanthin are more effective in NPQ, as they have the least discrepancy between theoretical and actual Φ_PSII_ (**Figures 3A–E**). Therefore, in sub-saturating conditions, such as greenhouses or controlled light growth conditions, plants with only lutein or violaxanthin should have higher Φ_PSII_ than plants containing only zeaxanthin. It is worth mentioning that although *lute* plants had the same light tolerance as the *viol* mutants, *lute* is perhaps better protected as the ETR is significantly undermined (**Figure 6A**; Supplementary Figure S2A) in the *lute* plant, thus excitation pressure (1-qP) is higher (**Figure 6B**; Supplementary Figure S2B), yet it still has the same light tolerance as *viol* plants (**Figure 4**). Light tolerance could therefore be higher with the same pNPQ levels, if it were not for reduced photochemical capacities. Conversely, zeaxanthin expressing plants may be beneficial in fluctuating environments owing to their ability to delay the onset of photoinhibition (**Figure 5**). The use of an IMAGING-PAM to measure the whole leaf canopy during the pNPQ procedure or during a fluctuating environment could be an important future step to monitor photoinhibition at the canopy or whole plant level. A brief exploration into this technique (Supplementary Figures S4 and S5) demonstrates that *lut2* plants have the most heterogenic canopy, with a clear difference in between old and new leaves. Thus this could elucidate an age dependent role for lutein in photoprotection. This could also have been a contributing effect to the reduced ETR in the *lut2* plants, along with different growth conditions, and a different method of ETR analysis compared to that previously used (Dall’Osto et al., 2006), however, this canopy heterogeneity is beyond the scope of this paper. The results do support the conclusions of Carvalho et al. (2015), who found that younger leaves and the innermost leaves on the rosette are less phototolerant than the more mature outer leaves, and this is something that should be taken into consideration when calculating/predicting whole canopy yields.

Despite the vast research and the competing reports into which carotenoid is the ‘true quencher’, it appears that a ‘cocktail’ of carotenoids in their correct binding sites serves plants best in forming pNPQ, suggesting a strong structural effect of the molecules upon the LHCII complexes and overall antenna organization for the best light harvesting as well as photoprotective function. This better preserves the openness of RCII, thereby maintaining a high ΦPSII for most environments. This is unsurprising given that higher plants are the result, albeit unfinished, of millions of years of evolution in the green lineage to select the required carotenoids to fulfill the function of excess energy dissipation.

## Author Contributions

The authors declare no conflicts of interest. MW and AR designed the project. LD generated plant material used in the experiment. MW grew plants and performed all experiments. All authors contributed to the interpretation of results and writing of the manuscript. All authors approve the manuscript publication.

## Conflict of Interest Statement

The authors declare that the research was conducted in the absence of any commercial or financial relationships that could be construed as a potential conflict of interest.
