# Supplementary material for: An In Vivo Quantitative Comparison of Photoprotection in Arabidopsis Xanthophyll Mutants
Source: Front Plant Sci. 2016 Jun 21;7:841. doi: 10.3389/fpls.2016.00841 (PMC4914555; doi:10.3389/fpls.2016.00841)
Supplement: Supplementary file 1 [file Data_Sheet_1.PDF]

An *in vivo* quantitative comparison of photoprotection in *Arabidopsis* xanthophyll mutants

Maxwell A. Ware[1], Luca Dall'Osto[2] and Alexander V. Ruban[1]*

[1]School of Biological and Chemical Sciences, Queen Mary University of London, Mile End Road, London E1 4NS, UK

[2]Dipartimento di Biotecnologie, Università di Verona, 37134 Verona, Italy

*Correspondence
a.ruban@qmul.ac.uk

Supplementary Table 1

	**Genotype**	**% violaxanthin**	**% zeaxanthin**	**% lutein**
**Dark adapted (1-hr)**	WT	12.5	0	69
	lut2	76.5	0	0
	npq2	0	42.6	57.4
	viol	100	0	0
	lute	0	0	100
	zea	0	100	0
**Light adapted (800 µmol m^{-2} s^{-1})**	WT	14.7	15.7	54.5
	lut2	62.9	21.6	0
	npq2	0	65.6	34.4
	viol	100	0	0
	lute	0	0	100
	zea	0	100	0

Supplementary Table 1. HPLC was performed on intact chloroplasts in 100% methanol. Xanthophyll percentages were calculated as [mmol of a xanthophyll specie/(mmol of total xanthophylls)]*100. Chloroplast preparations were performed from three plants of each genotype, with results from a single representative experiment. Violaxanthin, zeaxanthin and lutein were the only xanthophylls used when expressing percentages, as these are the focus of this research and correspond to previous publications (Havaux & Niyogi, 1999).

Supplementary Material Fig. 1

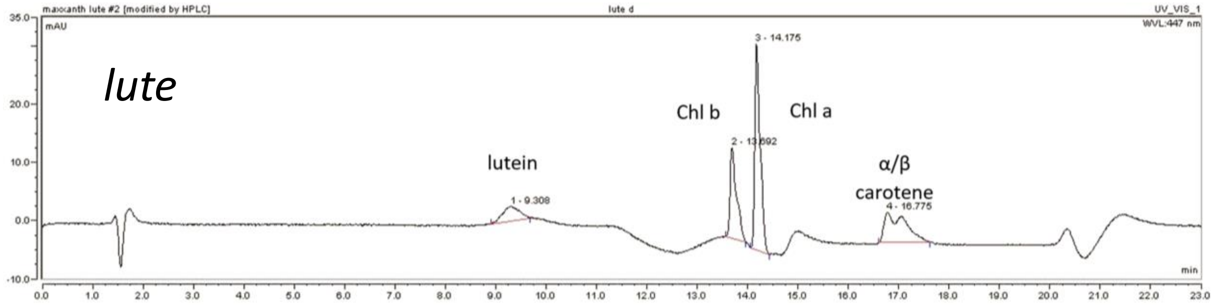

Supplementary Fig 1. Typical reverse-phase HPLC profile of chloroplasts from the *lute* plant, using a LiChrospher 100 RP-18 column and Dionex Summit chromatography system (Ruban et al. 1994). Carotenoid identification was performed in 100% methanol.

Supplementary Material Fig. 2

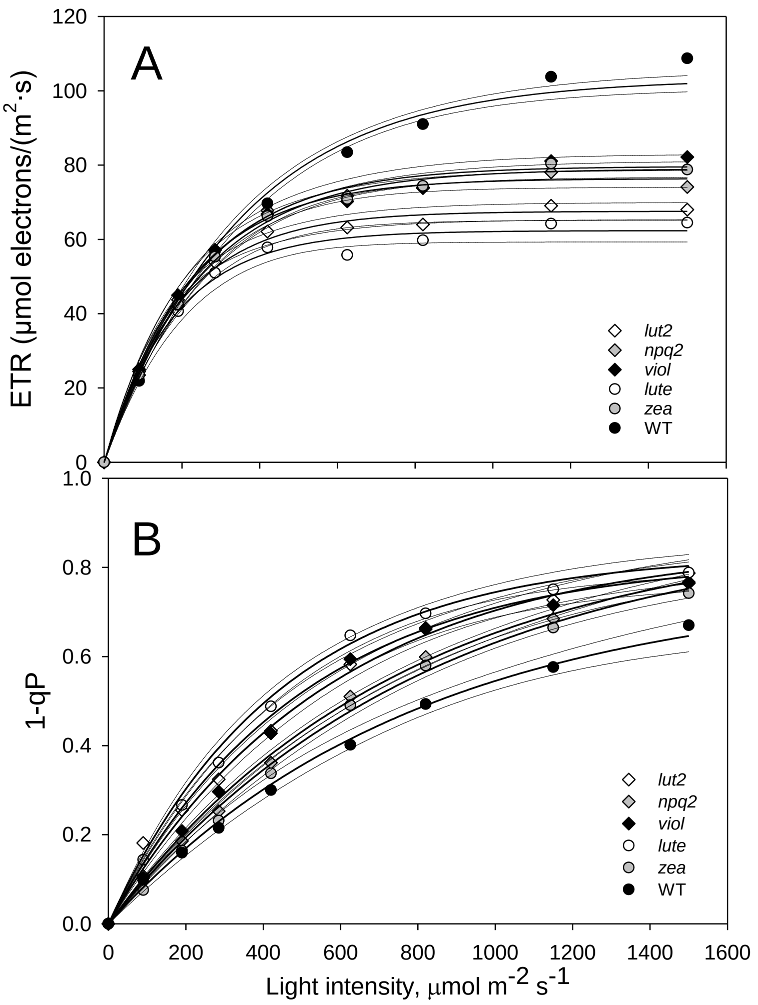

Supplementary Fig. 2A Electron transport rates (ETR) taken from the 2nd saturating pulse after each 5 min actinic light (AL) illumination period (See Fig. 1A). Regression analysis was performed using SigmaPlot12 (Exponential Rise to Maximum, Single 2 Parameter, f = a*[1-exp(-b*x)]). Data points are the average of 10 independent experiments (n=10). Fig. 6B represents the excitation pressure recorded at each 2nd saturating pulse after each 5 min actinic light (AL) illumination period (See Fig. 1A). Regression analysis was performed using SigmaPlot12 (Exponential Rise to Maximum, Single 2 Parameter, f = a*[1-exp(-b*x)]). Data points are the average of 10 independent experiments (n=10). *lut2* – no lutein, *npq2* – no violaxanthin and neoxanthin, *viol* – violaxanthin only, *lute* – lutein only, *zea* – zeaxanthin only, WT – wild type.

Supplementary Material Fig. 3

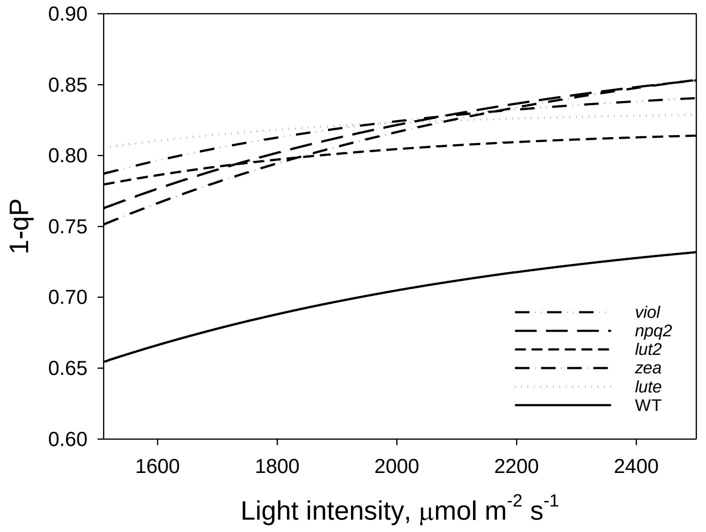

Supplementary Fig 3. An extrapolation of Fig. 6B, where the excitation pressure was recorded at each 2nd saturating pulse after each 5 min actinic light (AL) illumination period (See Fig. 1). Regression analysis was performed using SigmaPlot12 (Exponential Rise to Maximum, Single 2 Parameter, f = a*[1-exp(-b*x)]). *lut2* – no lutein, *npq2* – no violaxanthin and neoxanthin, *viol* – violaxanthin only, *lute* – lutein only, *zea* – zeaxanthin only, WT – wild type.

Supplementary Material Fig. 4

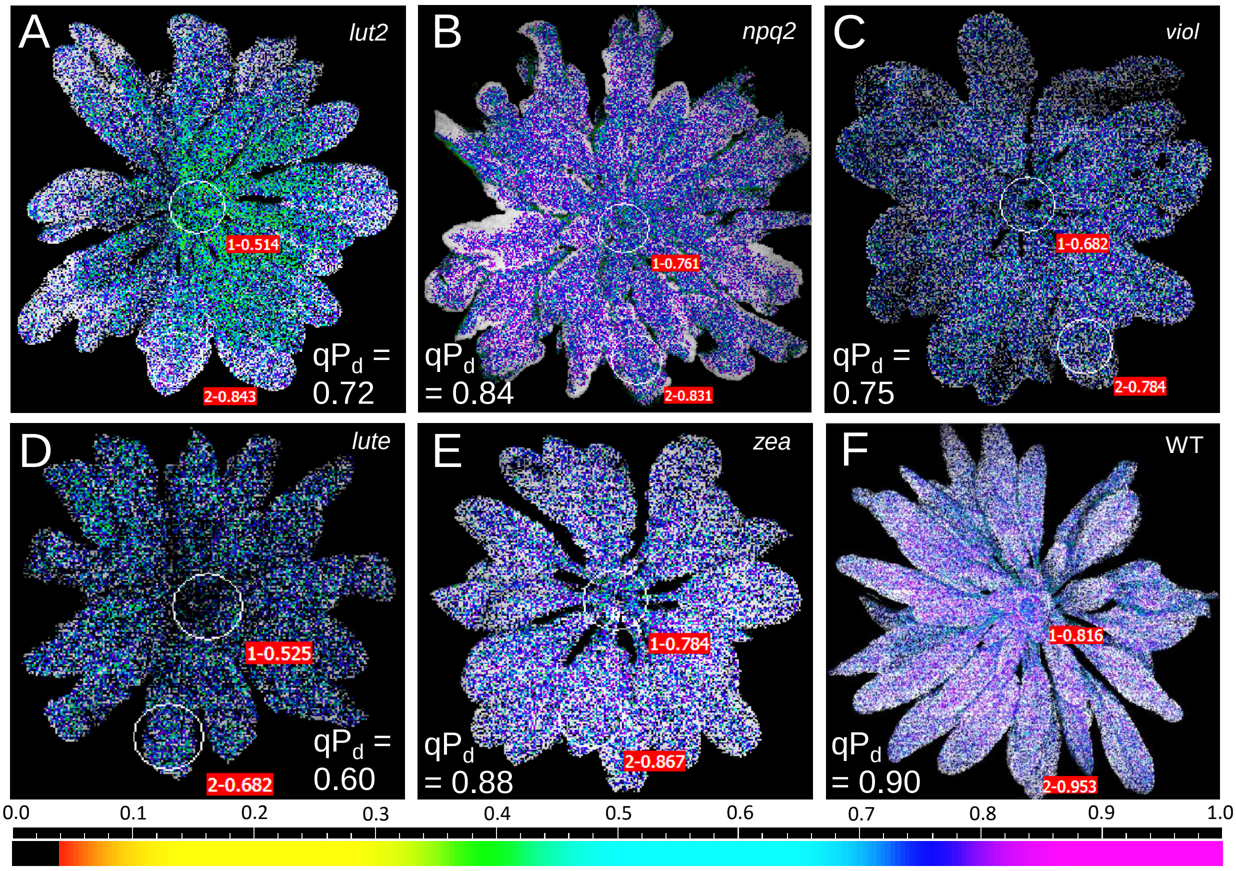

Supplementary Fig 4. pNPQ assessment procedure performed using an IMAGING-PAM (Walz, Effeltrich, Germany). Actinic light intensities of 0, 81, 186, 281, 396, 611, 801, 1076, 1250 µmol m^{-2} s^{-1} were used. Fluorescence image represents the average qP_d value for the whole leaf area at 1250 µmol m^{-2} s^{-1}. *lut2* – no lutein, *npq2* – no violaxanthin and neoxanthin, *viol* – violaxanthin only, *lute* – lutein only, *zea* – zeaxanthin only, WT – wild type. White circles are 9mm, indicating that WT plants are the largest at 50 days old and *lute* the smallest.

Supplementary Material Fig. 5

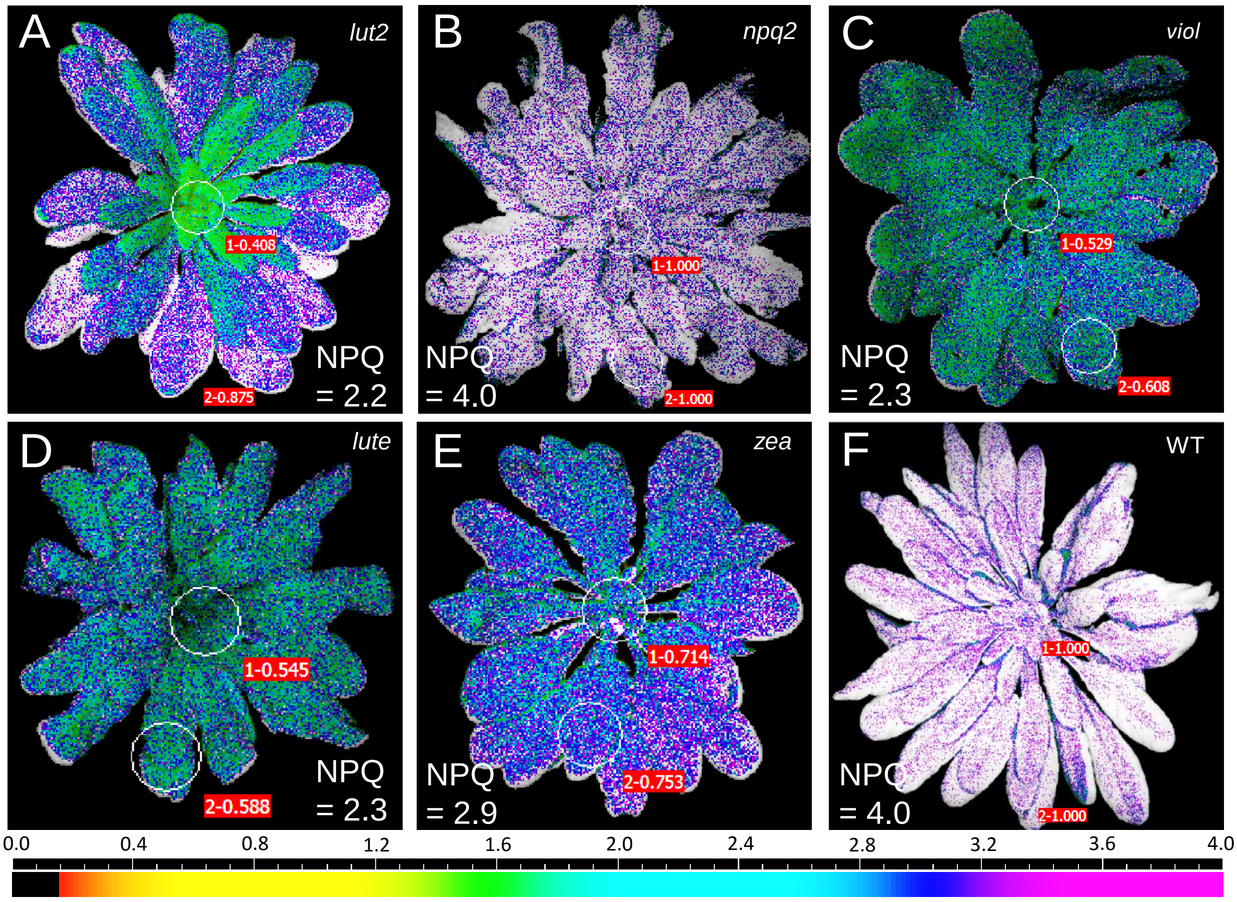

Supplementary Fig 5. pNPQ assessment procedure performed using an IMAGING-PAM (Walz, Effeltrich, Germany). Actinic light intensities of 0, 81, 186, 281, 396, 611, 801, 1076, 1250 µmol m^{-2} s^{-1} were used. Fluorescence image represents the average NPQ value for the whole leaf area at 1250 µmol m^{-2} s^{-1}. *lut2* – no lutein, *npq2* – no violaxanthin and neoxanthin, *viol* – violaxanthin only, *lute* – lutein only, *zea* – zeaxanthin only, WT – wild type. White circles are 9mm, indicating that WT plants are the largest at 50 days old and *lute* the smallest.
